# An online cursive handwritten medical words recognition system for busy doctors in developing countries for ensuring efficient healthcare service delivery

**DOI:** 10.1038/s41598-022-07571-z

**Published:** 2022-03-04

**Authors:** Shaira Tabassum, Nuren Abedin, Md Mahmudur Rahman, Md Moshiur Rahman, Mostafa Taufiq Ahmed, Rafiqul Islam, Ashir Ahmed

**Affiliations:** 1 Faculty of Information Science and Electrical Engineering, Kyushu University, Fukuoka, Japan; 2 Global Communication Center, Grameen Communications, Dhaka, Bangladesh; 3 Graduate School of Biomedical and Health Sciences, Hiroshima University, Hiroshima, Japan; 4 M A G Osmani Medical College, Sylhet, Bangladesh; 5 Medical Information Center, Kyushu University Hospital, Fukuoka, Japan

**Keywords:** Information technology, Scientific data, Health services

## Abstract

Doctors in developing countries are too busy to write digital prescriptions. Ninety-seven percent of Bangladeshi doctors write handwritten prescriptions, the majority of which lack legibility. Prescriptions are harder to read as they contain multiple languages. This paper proposes a machine learning approach to recognize doctors’ handwriting to create digital prescriptions. A ‘Handwritten Medical Term Corpus’ dataset is developed containing 17,431 samples of 480 medical terms. In order to improve the recognition efficiency, this paper introduces a data augmentation technique to widen the variety and increase the sample size. A sequence of line data is extracted from the augmented images of 1,591,100 samples and fed to a Bidirectional Long Short-Term Memory (LSTM) network. Data augmentation includes pattern Rotating, Shifting, and Stretching (RSS). Eight different combinations are applied to evaluate the strength of the proposed method. The result shows 93.0% average accuracy (max: 94.5%, min: 92.1%) using Bidirectional LSTM and RSS data augmentation. This accuracy is 19.6% higher than the recognition result with no data expansion. The proposed handwritten recognition technology can be installed in a smartpen for busy doctors which will recognize the writings and digitize them in real-time. It is expected that the smartpen will contribute to reduce medical errors, save medical costs and ensure healthy living in developing countries.

## Introduction

A global study reports that Bangladeshi physicians spend less than a minute on each primary consultation whereas physicians of Sweden spend 22.5 min^1^. This happens due to the massive population and limited number of physicians available for them in developing countries. The ideal doctor to population ratio recommended by World Health Organization (WHO) is 1:1000, while on the contrary, the ratio in Bangladesh is only 0.304:1000^2^. The consultation time of doctors includes listening to patients’ problems, inspecting test reports, writing a prescription, explaining the patients’ condition and giving advises. As they serve a large number of patients in a very short time, they are left with less time for writing a prescription. Thus, the handwriting on the prescription becomes cursive and indecipherable for the patients and pharmacists. As a result, the pharmacists misread the prescriptions due to the similar appearances or sounds of thousands of medication names^3^ and end up providing the patients with wrong medicines.

This research conducted an online survey to understand the current state of handwritten prescription usage in the medical practice of Bangladesh. The survey reports that 97.1% of Bangladeshi doctors still generate handwritten prescriptions. According to Bhuiyan et al.^4^, incompetency of understanding doctors’ handwritten prescriptions is an obstacle for getting quality health services. The difficulty of reading these prescriptions often causes adverse medical consequences such as selecting wrong medicine, improper number of dosage, and even death. National Academies of Science Institute reports that 7000 deaths occur in the US due to the sloppy handwriting of doctors’^5^. Doctors can be trained to write legible prescriptions, but they get a very short period of time to serve each patient.

This paper proposes a machine learning approach to assist the doctors by recognizing cursive handwriting of doctors and converting them into readable digital prescriptions. The handwriting recognition system consists of several steps: handwritten data collection, data prepossessing for simplification, increasing data samples using data augmentation, and building a machine learning model for predicting doctors’ handwriting.

Bangladeshi prescriptions are a mixture of Bangla and English words with Latin abbreviations of medical terms^4^. Due to the unavailability of enough Bangladeshi prescriptions online, this research started its journey by creating a ‘Handwritten Medical Term Corpus’. Initially, a medical words corpus is created using the 8,324 Bangladeshi prescriptions of the Portable Health Clinic (PHC). PHC is a remote healthcare system which provides affordable and sustainable health services to the base of the pyramid population of developing countries^6^^7^ to ensure universal healthcare coverage targeting rurality, poverty, and disability^8^. The PHC data has been used for predicting health status of the existing patients^9^^10^ in terms of healthcare cost reduction^11^, understanding consumer behavior^12^. However, none of these works considered the doctors’ role in the PHC system.

The ultimate performance of a remote healthcare system depends on the decision made by the doctor. As mentioned earlier, 97% of Bangladeshi doctors still write handwriting prescriptions. The trend is changing to digital prescriptions. The analog prescriptions are not archived in digital forms and are difficult to search for previous medical history. In order to create real-time digital prescriptions, a real-time handwriting recognition system is necessary. At the first step, a ‘Medical Term Corpus’ is needed. We have created a corpus considering the most used words that appeared in the PHC prescriptions. The corpus contains 480 medical-related words (English: 320 and Bangla:120). Afterward, the handwriting of 39 healthcare professionals of these 480 words are collected for the recognition purpose. Thus, the ‘Handwritten Medical Term Corpus’ has 17,431 handwritten instances.

Recognizing different types of handwriting requires a large dataset collected from various sources which is both costly and time-consuming. One way to deal with this problem is data augmentation^13^. This paper proposes a new data augmentation technique - Rotate, Shift, and Stretch (RSS) to generate multitudes of handwriting variations. RSS method takes each stroke of a handwritten word and creates new data by updating the coordinates. After applying RSS, the extended dataset has 1,591,100 samples. For predicting the handwritten words, Bidirectional LSTM model is used due to the recent popularity of Recurrent Neural Network (RNN) in the area of handwriting recognition. According to Zhang et al.^14^, sequence data can contain rich details of handwriting than static image-like representations.

The proposed handwritten technology can be used in a smartpen, specifically designed for doctors. The smartpen will digitize the handwriting of doctors into readable texts. The database will store each doctors’ writings individually to learn the unique pattern of writing of that particular doctor. This will lead the tool to recognize the handwriting of each doctor more efficiently. The smartpen will benefit the doctors by saving time and reducing typographical errors of digital prescriptions.

The rest of the article starts with a review on related researches in “Related work”, introduces a handwritten dataset of Bangladeshi doctors in “Handwritten medical term corpus”, demonstrates the steps of recognition methodology in “Methodology of handwriting recognition”, “Results and discussion” reports the results and findings including the idea of a smartpen, and finally, the conclusion is given in “Conclusion”.

## Related work

Over the last few decades, multitudes of deep learning approaches have been proposed for efficient handwriting recognition using several handwritten datasets of different languages. This section discusses similar research works in the following four sectors:

### Doctors’ handwriting dataset

Few online datasets are available to design a doctors’ handwriting recognition system. Dibyajyoti et al.^15^ introduced HP_DocPres dataset with 11,340 samples of handwritten and printed words collected from various medical prescriptions. This dataset is prepared to differentiate between handwritten and printed texts. However, the words are not labeled so they can’t be used to recognize the written words by doctors. Another doctors’ handwriting dataset is introduced by Farjado et al.^16^. This dataset contains 1800 images of 12 medicine names collected from 50 doctors from clinics and hospitals of Metro Manila, Quezon City, and Taytay, Rizal in the Philippines. However, this dataset is not suitable for recognizing doctors’ handwriting in Bangladeshi prescriptions due to the limited number of medical terms it contains and the region of data collected being different from our study region.

Although doctors’ handwriting dataset is scarce, there are multitudes of available handwriting datasets both for English and Bangla languages. IAM Dataset by the University of Bern^17^ is one of the most popular datasets with the largest handwriting collection in English. This dataset contains 13,353 images of handwritten lines of text created by 657 writers. A similar dataset in Bangla is the Bangla handwriting recognition dataset by Bappaditya et al.^18^ that has obtained 79,000 handwritten Bangla word samples written by 77 different writers. BanglaLekha-Isolated^19^ and ISI^20^ dataset comes with a vast number of handwriting samples of individual Bangla characters with numerals. Another popular dataset is CMATERdb1^21^ that has 100 handwritten Bangla pages and 50 handwritten English and Bangla combined pages with ground-truth annotations. However, these datasets do not contain doctors’ handwriting or any medical terms, hence might perform poorly in recognizing doctors’ handwriting.

### Offline handwritten character recognition: using image data as input

Automatic conversion of handwritten texts into images for recognition using Convolutional Neural Network (CNN) is called Offline Character Recognition^22^. Shahariar et al.^23^ proposed a lightweight CNN model for Bangla handwriting recognition. The model has 13 convolutional layers with 2 sub-layers. The sub-layers are joined together to pass through a max-pooling layer with one 0.25 weighted dropout layer. This model has attained 98%, 96.8% and 96.4% accuracy in BanglaLekha, CMATERdb, and ISI datasets. A modified LeNet-5 CNN model by Yuan et al.^24^ obtained an accuracy of 93.7% for uppercase and 90.2% for lowercase for the recognition for English Language characters. Yang et al.^25^ presented a path-signature feature method using deep CNN for identifying Chinese character writers. The method was 99.52% accurate with DropStroke data augmentation.

### Online handwritten character recognition: using time-series data as input

Online character recognition considers a sequence of times which is captured by the movements of a specialized pen. The recognition rate of the online system is more efficient and higher than the offline system^22^. RNN has recently been widely used in the area of handwriting recognition for showing better recognition performance. The RNNs work with sequence data of coordinates which contain vast information than static images^14^. Bappaditya et al.^18^ used bidirectional LSTM using 65,620 handwritten Bangla words dataset and has obtained 79% accuracy. Zhang et al.^14^ proposed a conditional RNN-based generative model combining LSTM and Gated Recurrent Units (GRU). The model is built for recognizing Chinese handwritten characters and has achieved 98.15% recognition accuracy. Farjado et al.^16^ used Convolutional RNN (CRNN) for recognizing doctors’ cursive handwriting which contained 13 convolutional layers followed by 3 bidirectional LSTM layers and has attained 72% accuracy. However, Achkar et al.^26^ reported obtaining 95% accuracy using the similar CRNN model with a different dataset for recognizing medical handwritten prescriptions.

### Handwriting recognition with data augmentation

In our previous work, SRP (Stroke Rotation and Parallel-shift) data augmentation technique was applied for expanding the doctors’ cursive handwritten dataset. However, the minimum accuracy of that system was only 68.0%^27^. For recognizing Bangla handwriting characters, Shahariar et al.^23^ applied three data augmentation methods on 10% of the dataset: shifted height and width, rotated images by 10 degrees, and zoomed in the images. Another data augmentation method named ‘DropStroke’ was used for Chinese character recognition. Chinese characters are very complex as they have many strokes. Thus, the DropStroke method randomly excludes several strokes and generates new handwritten characters using the combination of the remaining strokes^14^^25^. Hayashi et al.^13^ used a data augmentation technique using probability distribution for handwriting recognition. This method calculates probability distribution from the features related to the structure of the character. Then, it generates strokes based on the distribution and forms multitudes of new characters.

### Ethics approval

All the authors mentioned in the manuscript have agreed for authorship, read and approved the manuscript, and given consent for submission and subsequent publication of the manuscript.

### Consent to participate

The written informed consent was obtained from all subjects prior to collecting their handwritten samples in these studies.

### Consent for publication

The written informed consent was obtained from all subjects prior to collecting their handwritten samples in these studies.

## Handwritten medical term corpus

**Figure 1:**
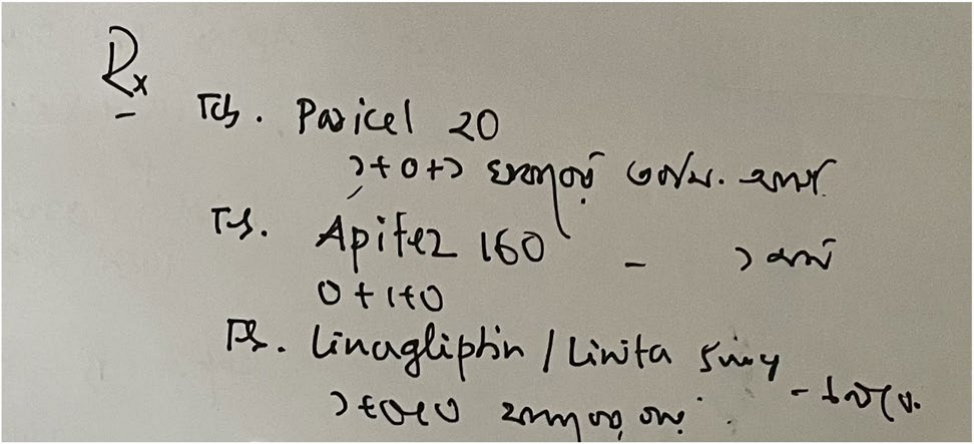
A sample image of Bangladeshi handwritten prescription.

In developing countries, Doctors’ handwriting becomes illegible as they have to serve a lot of patients in a short span of time. The writings get more difficult to read as Bangladeshi prescriptions are a mixture of different languages. A sample of Bangladeshi prescription is given in Fig. 1. Due to the shortage of available Bangladeshi prescriptions online, this research has introduced a handwriting prescription dataset collected from Bangladeshi doctors. This section describes the data collection and preparation process for creating a handwritten medical term corpus.

### Creating medical corpus from digital prescriptions

**Figure 2:**
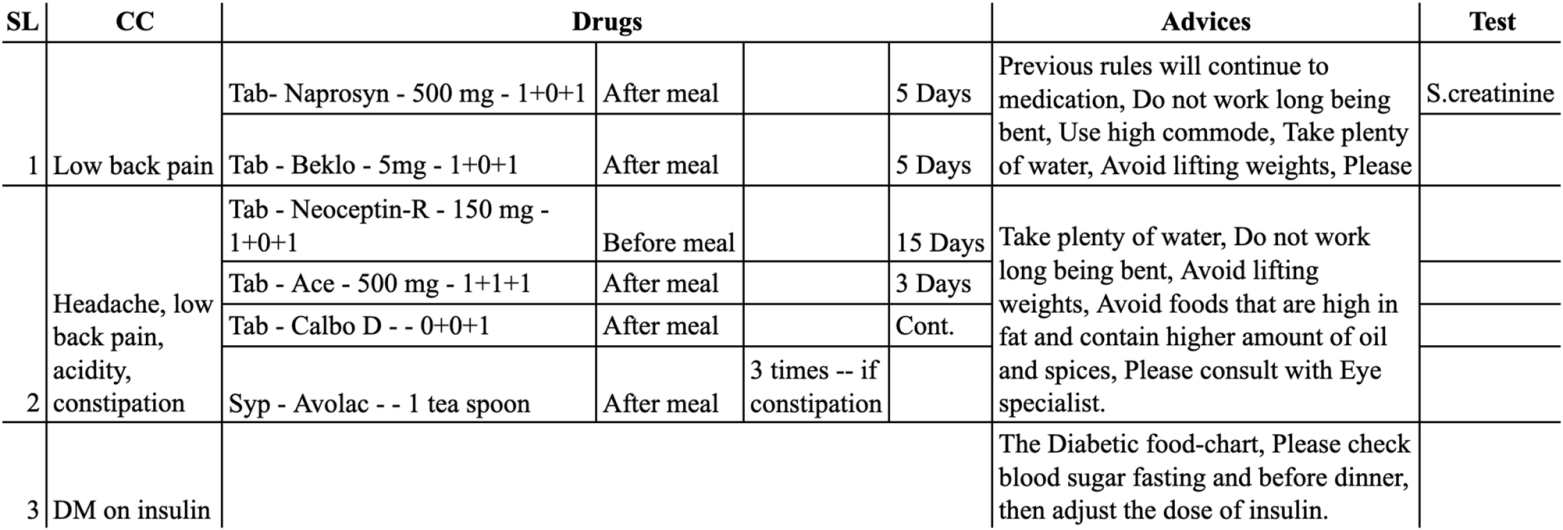
Sample of digital prescription of PHC.

Medical terms were collected from the remote healthcare prescription database of PHC. PHC system maintains an electronic journal of patients’ health records. There are major five categories of data in the journal: (1) registration data, (2) survey data, (3) clinical data, iv) conversation data, and v) prescription data. The foremost section of ‘Handwritten Medical Term Corpus’ is collected from the digital prescriptions of PHC. Figure 2 shows a sample of PHC prescription data.

**Figure 3:**
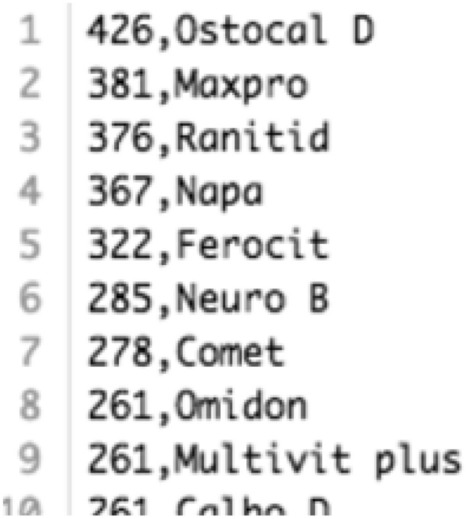
A segment from the Medical Term Corpus.

A total of 8324 digital prescriptions were found in the PHC database. Each prescription contained several columns such as symptoms, medicine names, advises. Initially, a corpus of medical terms is created using the most frequently appeared words in these columns. The corpus has selected 360 English and 120 Bangla words. These words are sorted according to the frequency of their appearance in the prescriptions, as shown in Fig. 3.

### Obtaining handwritten data via android application

**Figure 4:**
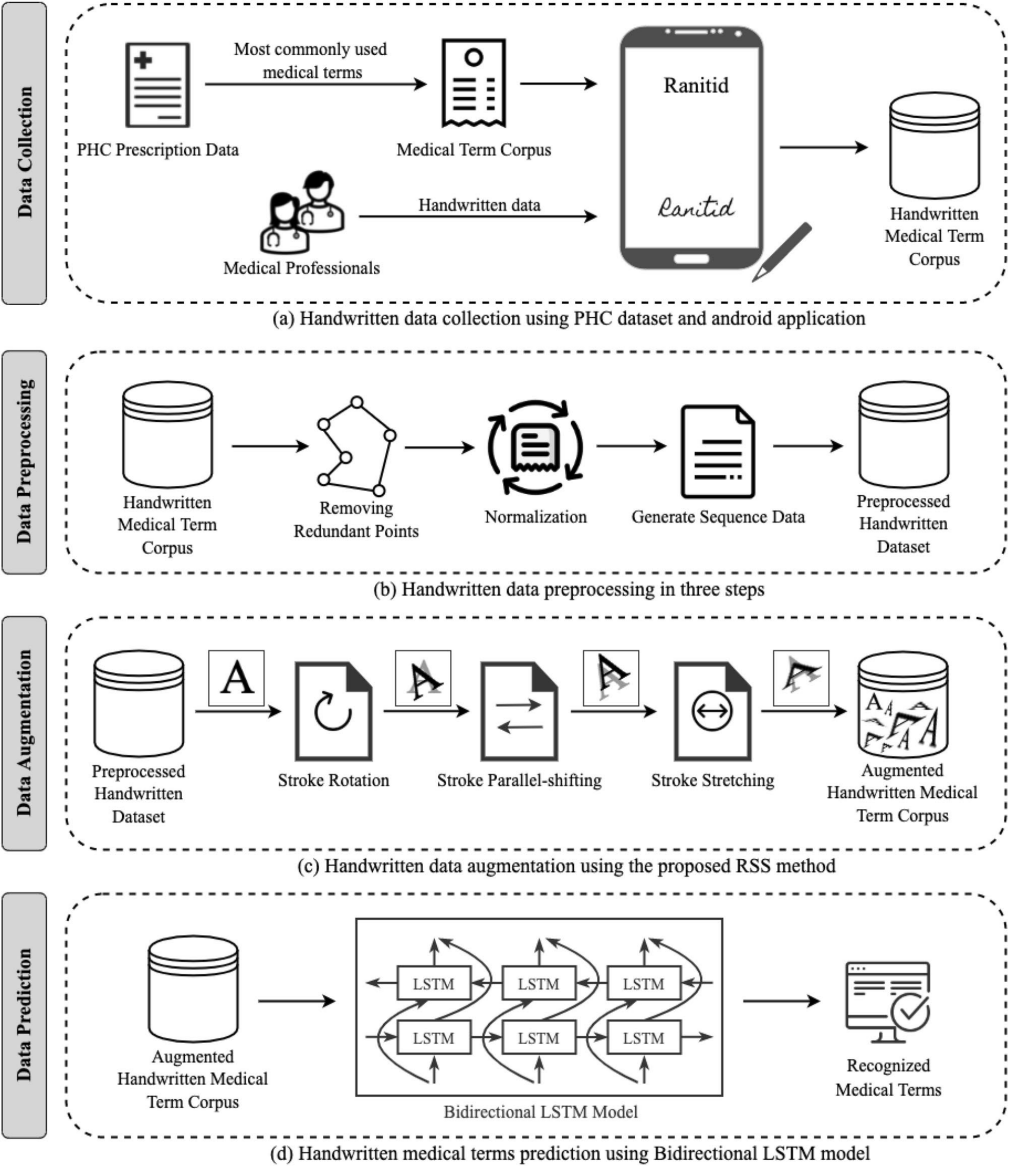
Overview of the handwritten medical terms recognition system.

A simple android app has been developed to obtain real handwriting data from doctors and medical professionals. The app displays medical words on the device screen one by one from the ‘Medical Term Corpus’. Then, the data providers write the corresponding words shown on the screen. Samsung Galaxy Tab S3 was used to capture data as it contains a stylus pen. The data providers write down on the screen using the stylus pen and the application stores the handwritten words in the database. Besides the writings, the application also receives detailed information such as pen movements (xy coordinates) and the status of the pen. Status indicates the state of the pen whether it is up or down. Collected information is stored in the database along with the writings as sequential data. The ‘Handwritten Medical Term Corpus’ also contains the original corpus data to use as truth value while training the machine learning model. The complete data collection process is shown in Fig. 4a.

### Dataset profile

There are 480 medical words (360 English and 120 Bangla) in the ‘Handwritten Medical Term Corpus’. These words are chosen based on the number of appearances in 8324 Bangladeshi prescriptions. The handwritings are collected from 39 medical professionals and doctors of Bangladesh. Due to receiving incomplete data from 12 data providers, 1,289 samples are missing in the dataset. Hence, the dataset has 17,431 handwritten instances of 480 medical-related words. All the data were collected by maintaining authenticity, security, and privacy of the data providers, and the experiments were performed in accordance with relevant guidelines and regulations.

## Methodology of handwriting recognition

After the data collection phase (Fig. 4a), the research is administered in three steps, as shown in Fig. 4b–d. First, the collected dataset is analyzed and preprocessed. Then, the proposed RSS (Rotation, Shift, and Stretch) data augmentation technique is applied to the preprocessed dataset to expand the number of instances. In this step, sequence line data is generated from the extended dataset. Finally, a bidirectional LSTM model uses the sequence data as input and predicts handwritten medical terms.

### Data preprocessing

Image-like representations provide very general information about the data. Whereas raw data is rich with information such as spatial and temporal details. These spatio-temporal information can be constituted in a sequence of variable length^14^, given in equation (1). Here, $x_i$ and $y_i$ denotes the xy-coordinates of pen movements and $s_i$ states the stroke number of any point *i*.

1 $$\begin{aligned} {[}[x_1,y_1,s_1],[x_2,y_2,s_2],\ldots ,[x_n,y_n,s_n]] \end{aligned}$$

As shown in Fig. 4b, the preprocessing is done in three steps. The images are simplified through removing nearby repetitive points and normalization. Then, a six-dimensional vector is extracted sequentially for each stroke to generate machine learning model input data. The three segments are described below:

#### Remove redundant points

Different styles of handwriting can be found even in a group of people with the same language. Different people follow different ways of writing such as small, regular, flat, cursive. Each writer creates distinct sampling points even if they are writing the same character. Thus, a general format for each character can be created by removing nearby redundant points for efficiently estimating the handwritten words. To remove all the redundant points from any handwritten word or character, consider a particular point $(x_i, y_i, s_i)$ where point *i* lies in the same stroke with its nearby points, as such $s_{i-1} = s_i = s_{i+1}$. There are two conditions to determine if point *i* should be removed:

(i) Distance between points: If the distance between two points *i* and $i-1$ is very small, then point *i* is removed. In the given Eq. (2), the threshold $T_{dist}$ = $0.005 * max(H,W)$, where H indicates the vertical and W indicates the horizontal widths of the handwriting text input place. Besides, two connecting points lying on a straight line is also be removed.
  2 $$\begin{aligned} \sqrt{(x_i-x_{i-1})^2 + (y_i-y_{i-1})^2} < T_{dist} \end{aligned}$$
(ii) Cosine similarity: Cosine similarity determines the similarity of an inner product space between two non-zero vectors. Point *i* is removed if similarity between two points *i* and $i-1$ is greater than the threshold cosine angle value. In the given Eq. (3), the threshold $T_{cos}$ is set to 0.99.
  3 $$\begin{aligned} \frac{\Delta {x_{i-1}}\Delta {x_i}+\Delta {y_{i-1}}\Delta {y_i}}{{(\Delta {x^2_{i-1}}+\Delta {y^2_{i-1}})}^{0.5} {(\Delta {x^2_i}+\Delta {y^2_i})}^{0.5}} >T_{cos} \end{aligned}$$

**Figure 5:**
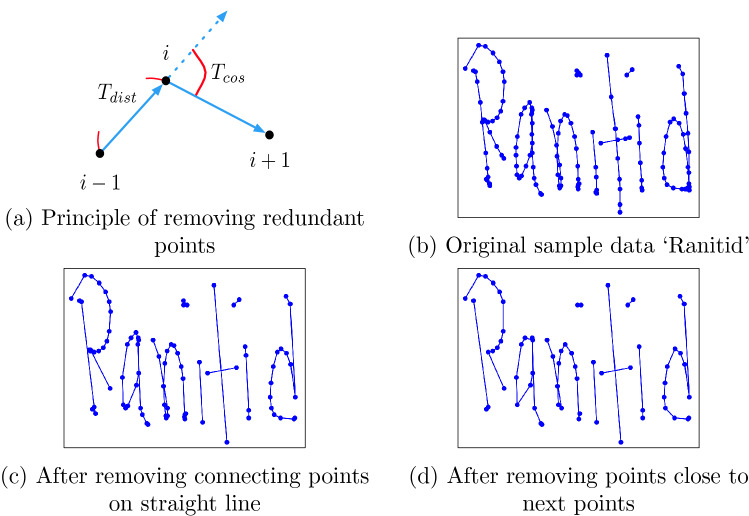
Remove redundant points from handwritten data.

Point *i* is considered as a redundant point if one of the given conditions is satisfied. After removing the redundant points, the shape of the handwritten character is well-preserved and each point of the recreated form contains more information^14^. An example of removing redundant points on a sample handwritten medical term is shown in Fig. 5.

#### Normalization

After removing the redundant points, the data is normalized for simplification. For *x* and *y* coordinates, the maximum $x_{max}$, $y_{max}$ and the minimum $x_{min}$, $y_{min}$ is calculated from each data point. Then, *x* as *X* and *y* as *Y* coordinates is normalized to $X_{nor}$ and $Y_{nor}$ using Eq. (4). Thus, the (*x*, *y*) coordinates data is scaled between the value of 0 and 1.

4 $$\begin{aligned} X_{nor} = \frac{X - x_{min}}{x_{max}-x_{min}} \end{aligned}$$

#### Generate sequence data

After the data preprocessing steps, this research has connected the normalized points to form straight lines. Then, a six-dimensional vector is generated from the straight line as $L_i$ with two connecting points *i* and $i+1$, as shown in Eq. (5).

5 $$\begin{aligned} L_i = [x_i, y_i, \Delta {x_i}, \Delta {y_i}, I(s_i=s_{i+1}), I(s_i \ne s_{i+1})] \end{aligned}$$

In the given equation, $x_i$ and $y_i$ are the xy-coordinates which states the starting position of a line. The direction of pen movements in x and y axis is denoted by $\Delta {x_i}$ and $\Delta {y_i}$. The last two expressions determine the status of the pen ([0, 1] indicates pen-up, [1, 0] indicates pen-down). The term $I(s_i=s_{i+1}) = 1$ indicates that the starting and ending points of the straight line lies on the same stroke. The last expression $I(s_i \ne s_{i+1}) = 1$ states that the line has moved to the next stroke. Thus, a new sequence of vectors $[L_1, L_2, \ldots , L_{n-1}]$ is formed from the (*x*, *y*, *s*) coordinates. This newly generated sequence is denoted as $[x_1,x_2,\ldots ,x_k]$ for simplification, where each $x_i$ represents one six-dimensional vector^14^.

### Data augmentation

This research has used data augmentation on the preprocessed data in order to increase the number of instances of ‘Handwritten Medical Term Corpus’. A new data augmentation approach named RSS (Rotation, Shift, and Stretch) is proposed in this article. RSS method expands data by rotation, shifting, and stretching the shape of character, as shown in Fig. 4c. This method is specifically designed to expand the variety of handwriting styles.

#### Rotate (stroke rotation)

**Figure 6:**
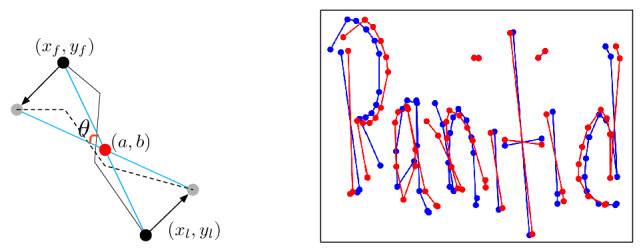
(**a**) Principle of rotation, (**b**) sample data after preprocessing (blue) and after rotation (red).

In stroke rotation, the middle point of a stroke (*a*, *b*) is determined using the starting point $(x_f, y_f)$ and ending point $(x_l, y_l)$ coordinates, as in Eq. (6). Then, all the points lying on the middle point of that stroke are rotated. The principle of the rotation process is shown in Fig. 6a.

6 $$\begin{aligned} (a, b) = (\frac{x_f+x_l}{2},\;\frac{y_f+y_l}{2}) \end{aligned}$$

Stroke rotation process uses Eq. (7) to rotate a point (*x*, *y*) in $\theta$ angle around the middle point of the stroke (*a*, *b*). The rotated point is (*X*, *Y*).

7 $$\begin{aligned} \left( \begin{array}{r} X-a \\ Y-b \end{array} \right) = \left( \begin{array}{rr} cos\theta &{} -sin\theta \\ sin\theta &{} cos\theta \end{array} \right) \left( \begin{array}{r} x-a \\ y-b \end{array} \right) \end{aligned}$$

This equation is applied to all of the points on the stroke to rotate the entire stroke around the midpoint of the stroke. Figure 6b is a sample example where the blue color is the original instance and the red color is the instance after rotation. This method is applied to the strokes with random values of angles in order to create new augmented data in different forms.

#### Shift (stroke parallel-shift)

**Figure 7:**
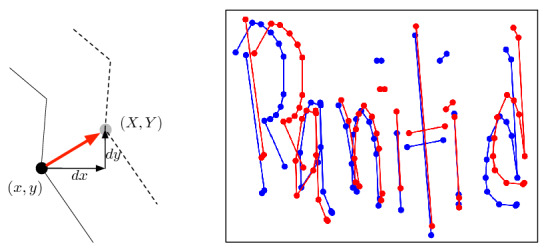
(**a**) Principle of shifting, **b** sample data after preprocessing (blue) and after Shifting (red).

In this second method, all of the points on the stroke are added to a constant number (*x*, *y*) in order to shift the strokes in parallel. The principle of parallel-shifting is given in Fig. 7a. One certain point (*x*, *y*) is shifted to a new point (*X*, *Y*) following Eq. (8).

8 $$\begin{aligned} (X, Y) = (x+dx,\;y+dy) \end{aligned}$$

The entire stroke is shifted by (*dx*, *dy*) after applying this equation to every point on the stroke. Figure 7b is a sample example where the blue color is the original instance and the red color is the instance after shifting. This method is applied to the strokes with random values of *dx* and *dy* in order to create new augmented data in different forms.

#### Stretch (stroke stretching)

**Figure 8:**
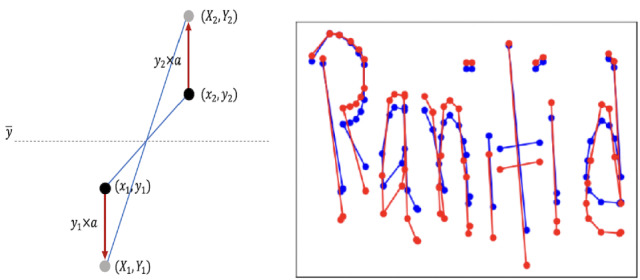
(**a**) Principle of stretching, (**b**) sample data after preprocessing (blue) and after stretching (red).

When a writer writes very quickly and roughly, the letters may be stretched vertically or horizontally. Hence, the stroke stretching method is proposed which takes all the strokes of a word and stretches the strokes to change the ratio of the handwritten word. The principle of this method is shown in Fig. 8a. First, the average value of the y-coordinates is calculated for all the points of a stroke as the reference value *Y*. If a certain point $(x_i, y_i)$ has larger y-coordinate that *Y*, then the value of $y_i$ is multiplied by $(1 + r)$, otherwise it’s multiplied by $(1 - r)$, shown in Eq. (9). Here, *r* is the changing ratio rate and is set to 0.02.

9 $$\begin{aligned} y_i = {\left\{ \begin{array}{ll} y_i \times (1 + r),&{} \text {if } Y < y_i\\ y_i \times (1 - r),&{} \text {otherwise} \end{array}\right. } \end{aligned}$$

However, when the writing is stretched, the points do not overlap and do not break the shape of the character. Figure 8b is a sample example where the blue color is the original instance and the red color is the instance after stretching. According to the findings of this research, this is an effective method for identifying rough handwriting.

### Regenerating sequence data

The original dataset contains six-dimensional vectors for all the instances. After data augmentation, the vectors need to be regenerated for the new augmented images by updating the parameters. For the rotated images, the values are updated by adding $\theta$ with $\Delta {x_i}$ and $\Delta {y_i}$, Eq. (10). To update the vector for parallel-shifting, *dx* and *dy* is added to $x_i$ and $y_i$, Eq. (11). Finally, the values of $x_i$ and $y_i$ are multiplied with *r* for the stretched images, Eq. (12). However, the values of $I(s_i=s_{i+1})$ and $I(s_i \ne s_{i+1})$ remain unchanged.

10 $$\begin{aligned}{}&\begin{aligned} L_i = [x_i,\;y_i,\;\Delta {x_i} + \theta ,\;\Delta {y_i} + \theta ,\;\\I(s_i=s_{i+1}),\;I(s_i \ne s_{i+1})] \end{aligned} \end{aligned}$$

11 $$\begin{aligned}{}&\begin{aligned} L_i = [x_i + dx,\;y_i + dy,\;\Delta {x_i},\;\Delta {y_i}\;\\I(s_i=s_{i+1}),\;I(s_i \ne s_{i+1})] \end{aligned} \end{aligned}$$

12 $$\begin{aligned}{}&\begin{aligned} L_i = [x_i \times r,\;y_i \times r,\;\Delta {x_i},\;\Delta {y_i},\;\\I(s_i=s_{i+1}),\;I(s_i \ne s_{i+1})] \end{aligned} \end{aligned}$$

The RSS data augmentation is designed specifically targeting handwriting data as the operations are done by updating the strokes. It can also be used for other datasets if the data are obtained as time series—a sequence of coordinates. If the time series data is converted into images, it can also be used for the expansion of offline characters. The augmented data is stored in the ‘Augmented Handwritten Medical Term Corpus’ dataset, as shown in Fig. 4c.

### Machine learning model: bidirectional LSTM

Handwriting contains multiple strokes with several points. Writing style, speed, order, shape of the character varies from person to person which information is difficult to achieve from static images. Hence, this research has dealt with raw sequential data rather than generating image-like representations in order to get rich information about doctors’ handwriting.

**Figure 9:**
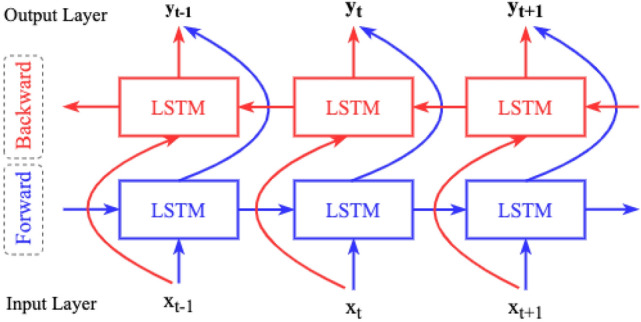
Concept of bidirectional LSTM.

This research has used Bidirectional LSTM to develop a complete end-to-end recognition system by operating the sequence data extracted from the line data of the augmented handwritten dataset, as shown in Fig. 4d. Bidirectional LSTM uses both past and future inputs for prediction, as shown in Fig. 9, whereas the original LSTM considers only past inputs^28^. In this research, the machine learning model has used both past and future line data to calculate parameters and predict handwritten medical words.

The model architecture for this research is developed using Keras - a neural network library of python. The maximum length of each data is set to 260 by padding zeroes by the end of the instances. There are 300 hidden LSTM layers with corresponding pooling layers. In order to avoid over-fitting, Dropout is used between pooling layer and dense layer^29^. As the model learns the same data many times due to using data augmentation, the Early Stopping method is also used to circumvent overfitting^30^. The Bidirectional LSTM model has the following parameters:

- Activation function: Softmax^31^
- Batch size: 512^32^
- Learning rate: 0.001^32^
- Number of epochs: 5^32^
- Loss function: Categorical cross-entropy^33^
- Optimization function: Adam^34^
- Dropout: 0.3^29^

## Results and discussion

The ‘Handwritten Medical Term Corpus’ contains 17,431 handwritten samples of 480 medical words. The writings are obtained from 39 medical professionals. Among the 39 sets, there are 27 complete sets of instances due to receiving incomplete data from 12 writers. Three sets of 480 words are randomly selected from the complete sets as test data. Thus, the train data has 15,911 and the test data has 1440 handwritten samples. The data augmentation methods are applied only to the train data. Thus, the Bidirectional LSTM model is trained using the extended sequence data and is evaluated based on its performance on the test set.

### Results

**Figure 10:**
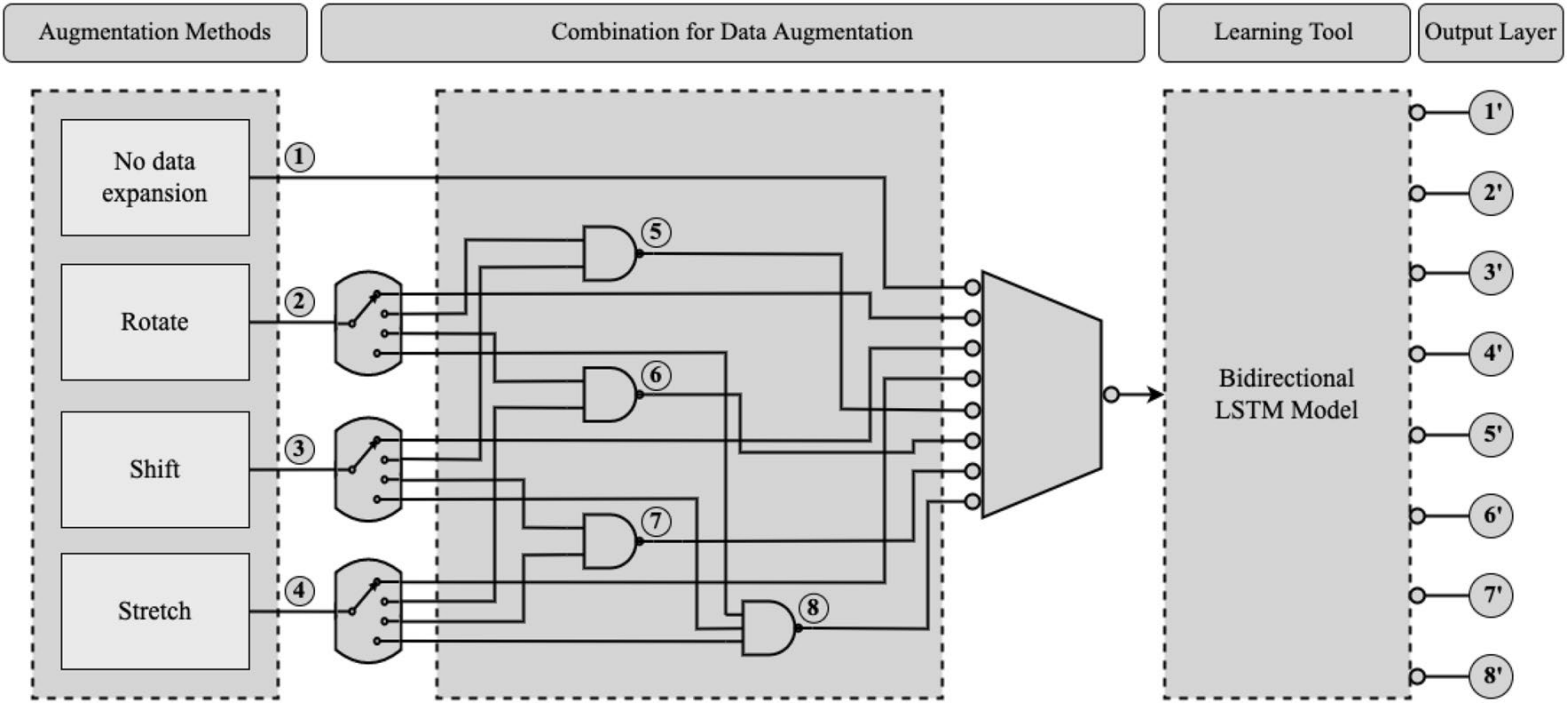
Training bidirectional LSTM model by different combination of data augmentation methods.

This research has performed eight sets of experiments by training the model with eight different mixtures of handwritten data. Figure 10 demonstrates the applied eight datasets expanded using different combination of data augmentation methods. The performance of the model is evaluated based on its accuracy on the same test set. The findings from the model evaluation are as follows, given in Table 1:

1. **No data expansion: ** First, the bidirectional LSTM model is trained on the original ‘Handwritten Medical Term Corpus’ with 15,911 instances without applying any data augmentation method. This case has attained the lowest accuracy.
2. **Rotate: ** This case has trained by model by applying only Rotation data augmentation method. Rotation is applied 100 times on each instances and the data size becomes 1,591,100. This experiment has achieved high maximum accuracy, but the lowest and unacceptable minimum accuracy which is only 3.33%.
3. **Shift: ** Shifting data augmentation is used 100 times on the train data. It has shown overall good performance in all the above three measurements.
4. **Stretch: ** Stretching data augmentation is applied similarly on the train data and the expanded data size is 1,591,100. This experiment has achieved similar results to Shifting data augmentation.
5. **Rotate + Shift: ** This experiment has combined the Rotate and Shift data augmentation methods. First, each instance is rotated ten (10) times and the data size becomes 159,110. Then, Shift method is performed for another ten (10) times on the expanded instances. Thus, the final data size gets 1,591,100 instances. The maximum accuracy is quite high but the minimum accuracy is comparatively low.
6. **Rotate + Stretch: ** The combined Rotation and Stretching methods are applied in this experiment in the similar way. This case has attained the highest maximum accuracy. However, the average and minimum accuracy is low comparing to the other cases.
7. **Shift + Stretch: ** Shifting and Stretching data augmentation methods are combined in this case. The data size is 1,591,100 by applying the methods ten (10) times each. This experiment has also accomplished overall considerable accuracy, but better results were observed when these two methods are applied individually on the training data (case **3** and **4**).
8. **Rotate + Shift + Stretch (RSS): ** Finally, this is the proposed data augmentation technique of this research. It applies all the three methods on the training data one by one. First, the instances are rotated five (5) times. Then, shifting is performed for another five (5) times on the expanded 79,555 instances. The data size becomes 397,775 where we have applied the stretch method for four (4) times. Thus, this research introduces the ‘Augmented Handwritten Medical Term Corpus’ which contains 1,591,100 handwritten medical term samples. The Bidirectional LSTM model with RSS data augmentation has achieved the highest average and minimum accuracy. It could not reach the highest maximum accuracy but the accuracy never plunged under 92.1%. Thus, this experiment can be considered as the best possible method for recognizing doctors’ cursive handwriting.

**Table 1:** Data augmentation performance evaluation on Handwritten Medical Term Corpus.

SL.	Method	Model accuracy (%)			Data size
		Avg	Max	Min	
1.	No data expansion	73.4	90.8	19.0	15,911
2.	Rotate	83.8	95.6	3.33	1,591,100
3.	Shift	91.3	93.9	87.8	1,591,100
4.	Stretch	91.4	95.7	87.5	1,591,100
5.	Rotate + Shift	89.5	95.4	68.0	1,591,100
6.	Rotate + Stretch	90.0	**95**.**9**	77.4	1,591,100
7.	Shift + Stretch	88.8	94.2	78.0	1,591,100
8.	Rotate + Shift + Stretch	**93**.**0**	94.5	**92**.**1**	1,591,100

The bold values represent the highest accuracy achieved by each data augmentation method.

### IoT smartpen for doctors: an application example

**Figure 11:**
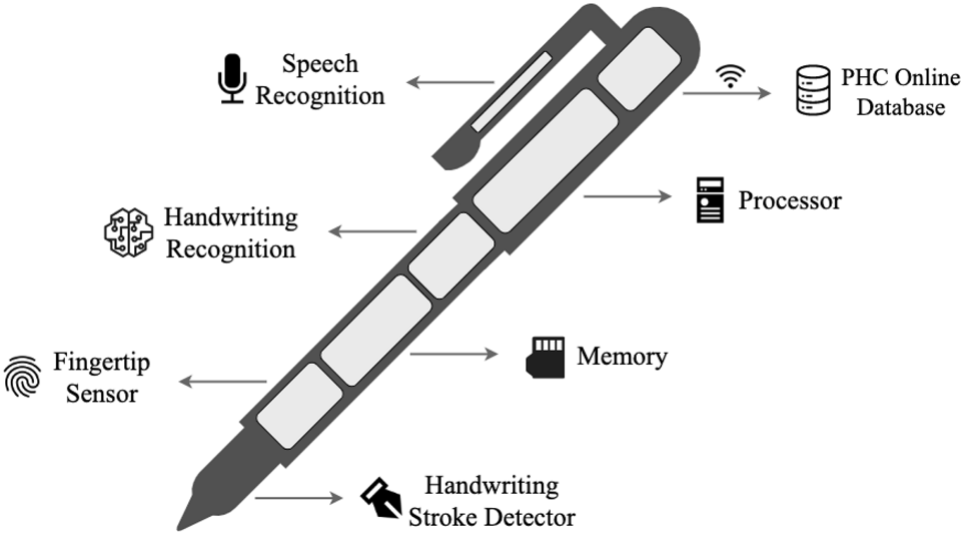
IoT smartpen design for doctors.

The handwriting recognition tool can be installed in a doctor’s smartpen, which is an ongoing work at our research institution. As shown in Fig. 11, the smartpen has seven major modules. The same recognition tool can also be installed in tablet PCs. The **Handwriting Stroke Detector** contains a sensor in the nib of the pen to collect words written by a doctor. The **Fingertip Sensor** will recognize the authorized user of the pen so that unauthorized person can not use the pen. The **Memory** will store all the prescriptions so that the doctor can easily find previous health records. It can also store new patterns of words written by the doctor.

In this way, the data set will get larger day by day. The system will also able to capture the unique handwriting habit of that particular doctor. The **Handwriting Recognition** tool will recognize the words written by the doctor and convert them into text to store it to the memory. A copy of the prescription will also be stored in **PHC Online Database** that can be accessed by the patient, authorized pharmacy and family members. The **Speech Recognition** will be used as a sound recorder to generate prescriptions from doctors’ speech through voice recognition. However, that module will use a different technology which is not part of this particular research of handwriting recognition.

As mentioned above, the smartpen is still in the concept level where our handwriting recognition technique can be applied. This smartpen will handle sensitive information such as patients’ identification, medical history, doctors’ profiles including bio-metric information. The smartpen development process will follow relevant privacy security guidelines such as data privacy for m-health patients^35^ , location based privacy^36^ , privacy protection of health records from search engines^37^.

## Conclusion

The objective of this research has been to recognize doctors’ handwriting and digitize the prescriptions in real time. Towards this goal, this paper contributes in three steps- (a) develop a medical term corpus (b) introduce a unique data augmentation technique and (c) use a machine learning approach for final recognition. It also compares the recognition accuracy in different augmentation stages.

The machine learning approach was designed for recognizing particularly doctors’ cursive handwriting and converting them into digital printed texts. A dataset named ‘Handwritten Medical Term Corpus’ was created from digital prescriptions of PHC that contains 17,431 handwritten texts of 480 Bangla and English medical-related words. A data augmentation method RSS was proposed for enriching the variety of doctors’ handwriting. RSS method expanded the data set to 1,591,100 instances which was also introduced in this paper named ‘Augmented Handwritten Medical Term Corpus’. Bidirectional LSTM model was used to create an online character recognition system for predicting doctors’ handwriting. This research performed eight experiments on the handwritten data set and achieved 93.0% average accuracy (max: 94.5%, min: 92.1%) using Bidirectional LSTM and RSS data augmentation. This accuracy was 19.6% higher than the recognition result with no data expansion.

The current accuracy needs to be improved. The proposed recognition methodology can be implemented in a smartpen for doctors. A brief system architecture of the proposed smartpen is introduced. Doctors will use the smartpen for writing and the tool will automatically convert the handwriting texts into digital prescriptions. Apart from the proposed method for data augmentation, other representative computational intelligence algorithms can be used to solve similar problems like Monarch Butterfly Optimization (MBO)^38^, Earthwarm Optimization Algorithm (EOA)^39^, Elephant Herding Optimization (EHO)^40^, Moth Search (MS) Algorithm^41^, and Harris Hawk Optimization (HHO)^42^. More samples will be collected and will be automatically stored in the corpus. Doctors personal handwriting habit will also be captured. More samples will improve the recognition accuracy.

## Data Availability

The data and materials are available in the Social Technology Lab page at http://socialtech.gramweb.net/media-archive/codes.

## Code availability

All codes are available in the Social Technology Lab page at http://socialtech.gramweb.net/media-archive/codes.
